# 

**Published:** 1848-04

**Authors:** 


*Ranking's Abstract.*—No. 6 of this valuable work has been promptly issued by the enterprising publishers, Lindsay and Blakiston, of Philadelphia. We need not repeat our recommendations of the work. Every intelligent practitioner buys it as a matter of course.

---

*To Correspondents.*—Communications have been received from Drs. Ganson, Green, and Meachem, which will appear in our next.

